# Living Safely With Bats: Lessons in Developing and Sharing a Global One Health Educational Resource

**DOI:** 10.9745/GHSP-D-22-00106

**Published:** 2022-12-21

**Authors:** Stephanie Martinez, Ava Sullivan, Emily Hagan, Jonathan Goley, Jonathan H. Epstein, Kevin J. Olival, Karen Saylors, Jason Euren, James Bangura, Sijali Zikankuba, Mohamed Moctar Mouliom Mouiche, Alpha Oumar Camara, James Desmond, Ariful Islam, Tom Hughes, Supaporn Wacharplusadee, Veasna Duong, Nguyen Thi Thanh Nga, Brian Bird, Tracey Goldstein, David Wolking, Christine K. Johnson, Jonna AK Mazet, Sarah H. Olson, Amanda E. Fine, Marc Valitutto, William B. Karesh, Peter Daszak, Leilani Francisco, the PREDICT Consortium

**Affiliations:** a EcoHealth Alliance, New York, NY, USA.; b Labyrinth Global Health, St. Petersburg, FL, USA.; c Metabiota, Inc., San Francisco, CA, USA.; d One Health Institute, University of California, Davis, Davis, CA, USA.; e Sokoine University of Agriculture, Morogoro, Tanzania.; f School of Veterinary Medicine and Sciences, University of Ngaoundéré, Ngaoundéré, Cameroon.; g Expert Surveillance Interface H/A/E FAO-ECTAD, Conakry, Guinea.; h Liberia Chimpanzee Rescue and Protection, Monrovia, Liberia.; i Conservation Medicine, Selangor, Malaysia.; j Thai Red Cross Emerging Infectious Diseases Clinical Center, King Chulalongkorn Memorial Hospital, Bangkok, Thailand.; k School of Global Health, Chulalongkorn University, Bangkok, Thailand.; l Virology Unit, Institut Pasteur du Cambodge, Pasteur Network, Phnom Penh, Cambodia.; m Wildlife Conservation Society, Viet Nam Country Program, Hanoi, Vietnam.; n Wildlife Conservation Society, Health Program, Bronx, NY, USA.; o Smithsonian Institution, Washington, DC, USA.; p Henry Jackson Foundation, Bethesda, MD, USA.

## Abstract

As part of a public health behavior change and communication strategy related to the identification of a novel ebolavirus in bats in Sierra Leone in 2016, a consortium of experts launched an effort to create a widely accessible resource for community awareness and education on reducing disease risk. The resulting picture book, *Living Safely With Bats*, includes technical content developed by a consortium of experts in public health, animal health, conservation, bats, and disease ecology from 30 countries. The book has now been adapted, translated, and used in more than 20 countries in Africa and Asia. We review the processes used to integrate feedback from local stakeholders and multidisciplinary experts. We also provide recommendations for One Health and other practitioners who choose to pursue the development and evaluation of this or similar zoonotic disease risk mitigation tools.

## Abstract (precis)

The *Living Safely With Bats* picture book development process provides a model for collaboratively creating educational resources to combat zoonotic disease spillover risk. We discuss lessons learned from the process and future considerations for tool development and evaluation.

## Teaser

**Key Findings:**

- From the *Living Safely With Bats* book’s inception to distribution, the content development team engaged and collaborated with multilevel stakeholders from multiple countries on the diverse cultural contexts and local knowledge, attitudes, and behaviors regarding zoonotic diseases.
- The book development process provided insights on how to strategically adapt text and artwork targeted at community-level implementation to reinforce public health and wildlife conservation messages that could be tailored to audiences according to their risk compositions and local contexts.
- Integrating multisectoral feedback from experts in animal health, human health, and conservation at every facet of the project ensured that a One Health lens was applied throughout the development process.

**Key Implication:**

- Global research efforts should budget for both planned and potential projects that can translate findings into community-oriented, evidence-based risk communication materials. This includes providing support for evaluation activities.

## INTRODUCTION

Emerging infectious diseases (EIDs) are pressing challenges to global health, economies, and international health security.^1^ More than 60% of infectious diseases that are known in humans and 75% of all EIDs have an animal origin.^2^^,^^3^ Spillover of viruses from a wildlife host to the human population is a dynamic process that requires several factors to align, including host density and distribution and human exposure.^4^ Human exposure is also a dynamic process and can go beyond a single point-of-contact. Contact with wildlife, which could include direct and indirect contact, spatial overlapping, and proximity, is modified by demographic and qualitative factors.^5^

Novel zoonoses emerge as a result of human interactions with wild animals that are increasing in scale due to environmental changes and increasing globalization.^6^^,^^7^ These large-scale changes promote the spillover of novel viruses from animals to people as wild animal habitats are altered by deforestation, climate change, land-use change, and urbanization.^8^ Diverse human behaviors at the wildlife-livestock-human interface may increase the risk of spillover. With increasing human mobility, the disease is amplified and spread more rapidly, as illustrated by the 2013–2016 West Africa Ebola outbreak, the 2018 Democratic Republic of Congo outbreak, and the current coronavirus disease (COVID-19) pandemic.^9^

In 2009, the U.S. Agency for International Development (USAID) launched the Emerging Pandemic Threats program to lead a global effort in strengthening the capacity of key hotspot countries in preventing, detecting, and controlling zoonotic disease emergence.^10^^,^^11^ Under this program, the PREDICT project aimed to strengthen local capacities for the surveillance and early detection of viral threats in wildlife and to identify high-risk areas, human-animal interfaces, and human behaviors to inform future mitigation efforts.^10^^,^^11^ Teams of scientists and One Health practitioners from around the globe led the project, the most comprehensive One Health surveillance initiative to date.^10^ A One Health approach recognizes the interdependence of human, animal, and environmental health and seeks to practically bring together practitioners from these disciplines to collaboratively address issues pertaining to global public health and sustainability.

As part of its mandate, PREDICT targeted biosurveillance of high-risk taxa that are hypothesized to play an outsized role in spillover events between animals and humans.^12^ For example, filoviruses (e.g., Ebola and Marburg viruses), lyssaviruses (e.g., rabies virus), henipaviruses (e.g., Nipah virus), and beta coronaviruses (e.g., severe acute respiratory syndrome–related coronaviruses) have been associated with various bat species, and as such, bats were a key target for surveillance in the PREDICT project.^12^

During this biosurveillance project effort, the novel *Bombali ebolavirus* species (BOMV) was detected in bats inside people’s homes in Sierra Leone in May 2016.^13^ In response, regional health officials and community stakeholders requested support from experts from the PREDICT team in preventing panic around the discovery, given experiences from the previous Ebola epidemic. The project included a mixed-methods approach to conducting behavioral risk investigations in communities that had recently experienced local policy changes, messaging, and social impacts of Ebola.^14^ Preliminary findings from the behavioral risk investigation revealed a desire for locally actionable behavior change strategies related to living more safely alongside bats.

Preliminary findings from the behavioral risk investigation after discovering the Bombali ebolavirus revealed a desire for locally actionable behavior change strategies related to living more safely alongside bats.

Based on their high level of contact with bats, bat hunters were studied at the site where BOMV was first detected.^15^ This study identified multiple risk factors across knowledge, attitudes, beliefs, and practices related to bat contact within the bat hunter population at a community site in Bombali, Sierra Leone.^15^ Risk factors included a lack of concern related to close bat contact, despite the knowledge that bats were thought to be connected with past Ebola outbreaks.^15^ Additionally, the study describes hunters being bitten or scratched by bats, exposed to aerosols, and wearing little or no personal protective equipment during hunting.^15^ Given these findings, a pressing need existed for an evidence-based community education tool that could support regional public health efforts in Sierra Leone, Guinea, and Liberia. PREDICT launched the development of an evidence-based resource that could support communities in recognizing bats as a part of a healthy ecosystem while reducing contact that may lead to the spillover of zoonoses.

The resulting educational tool, a picture book titled *Living Safely With Bats*, was designed through a collaboration of subject matter experts and local stakeholders. This process provided rich insights on how to collaboratively build an effective One Health educational resource and strategically adapt text and artwork to reinforce public health and wildlife conservation messages that could apply in multiple national and cultural contexts. We present this report to support One Health practitioners who seek to engage in multisectoral approaches to build educational tools related to zoonotic disease transmission prevention.

## PICTURE BOOK DEVELOPMENT PROCESS

We describe the process that the multidisciplinary team of experts and stakeholders used to develop an evidence-based educational picture book, including how we structured the core messaging and integrated feedback.

**FIGURE 1:**
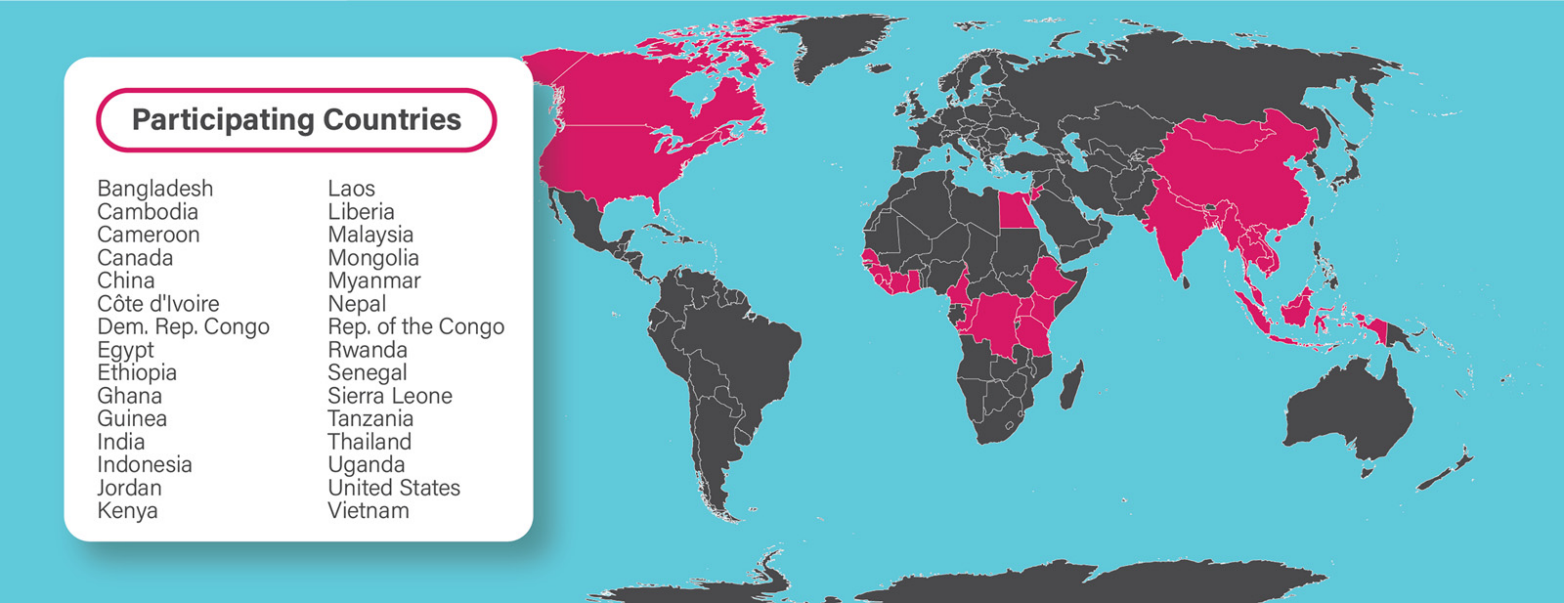
Map of One Health Collaboration for Developing Living Safely With Bats Book Included Teams From 30 Participating Countries

As an effort grounded in One Health, the book’s content needed to convey the interconnectedness of human health with wildlife and the environment. By highlighting the importance of bats and the critical role they play in the environment, we worked to foster discussion about personal risk mitigation while promoting positive information about the ecological importance of bats and discouraging destructive actions toward them that may lead to increased contact and environmental consequences. Thus, while the content promotes caution regarding direct contact with bats and their bodily fluids around the home and in the community, it also encourages individuals to take preventive measures that do not harm local wildlife, including seeking professional intervention when bats are in the home. Partner communities in Sierra Leone, Guinea, and Liberia were understood to be the first point of distribution due to the urgency of the BOMV discovery. However, since members of the PREDICT consortium anticipated that the resource would be used by other partner countries throughout Africa and Asia, we sought input from PREDICT partners in 30 countries (Figure 1). *Living Safely With Bats* was developed to be broadly applicable and accessible to all communities living near natural bat habitats, actionable at the household level, and adaptable to different country and specific local cultural contexts to be locally relevant.

We worked to foster discussion about personal risk mitigation while promoting positive information about the ecological importance of bats and discouraging destructive actions toward them that may lead to increased contact and environmental consequences.

### Engaging Stakeholders in Collaborative Development

**FIGURE 2:**
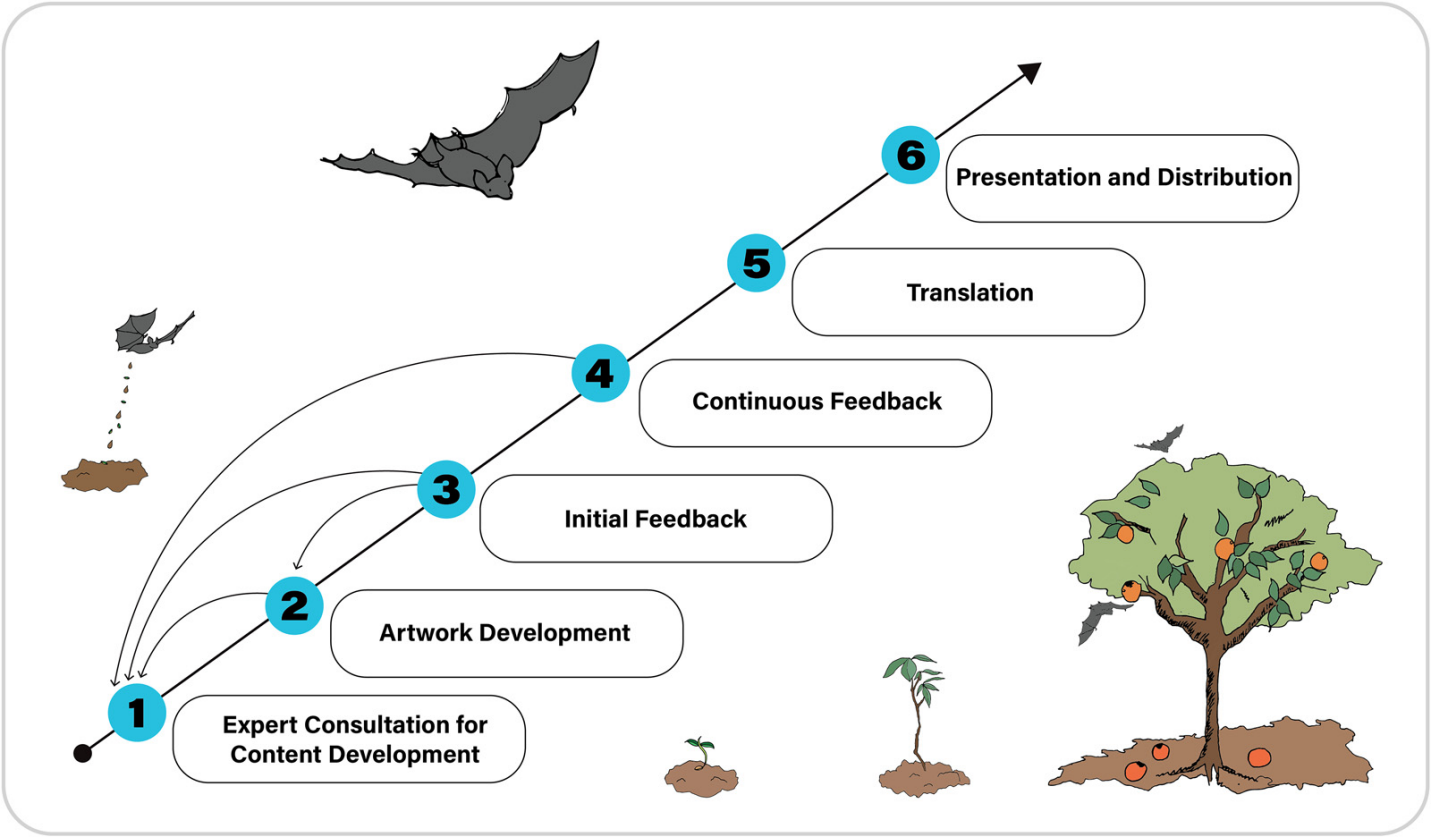
Key Steps in the Global Collaboration and Development of Living Safely With Bats Book

Although the picture book development was managed by a central technical and design team, *Living Safely With Bats* is a product of a multicountry, multidisciplinary collaboration. From inception to distribution, every effort was made to incorporate the cultural contexts, local knowledge, and technical expertise from within the consortium, which included social scientists, veterinarians, clinicians, ecologists, and epidemiologists from all the countries in which the book was intended for distribution. We detail the key steps in the consortium-wide effort that were taken to transform expert-driven suggestions into public-facing recommendations (Figure 2): (1) expert consultation for content development, (2) artwork development, (3) initial feedback, (4) continuous feedback, (5) translation, and (6) presentation and distribution.

#### Expert Consultation for Content Development

Initial consultations with subject matter experts in public health, animal health, conservation, bats, and disease ecology, including individuals who were a part of the outbreak response during the 2013–2016 Ebola outbreak and part of the team that detected the BOMV in bats in Sierra Leone, were essential to producing the first draft of the text.

#### Artwork Development

The team created regionally specific, digitized illustrations that corresponded to the key talking points for each page. The design team worked in close collaboration with the subject matter experts to create images that accurately reflected One Health messages and incorporate visuals that would be locally recognizable.

#### Initial Feedback

After the full first draft of the book was developed, the project’s Sierra Leone team collaborated with the USAID–funded Breakthrough ACTION project to garner feedback from community members, including elders, youth, and a traditional leader. The focus group collected initial impressions of the book, comprehension of and feelings associated with the content, and areas for improvement.

After an additional round of revisions to the book, feedback was solicited from more than 100 members of the PREDICT project consortium. This was a transformative step of the process, as experts, practitioners, and staff from participating countries gave critical feedback on messages that would be unclear or contextually inappropriate in their specific project settings. Reviewers also provided suggestions on ways to make the content more impactful for their audiences. All comments and suggestions, including those that were ultimately not incorporated, were recorded in a standardized comment tracking tool. This step also yielded a critical mass of requests for the development of a companion version of the book that could be used at common high-risk interfaces in Asia.

#### Continuous Feedback

After incorporating feedback from the consortium, the project continuously sought and incorporated feedback on the book’s text and imagery. The standardized comment tracker recorded the following: each suggestion, who suggested it, the content the suggestion applied to, notes such as additional resources to consult, and final comments from the design team regarding how the feedback had been incorporated. The tracker also included numeric ranking schemes to inform task prioritization and track action item completion. These rankings primarily served as a time management tool, with all comments eventually being addressed. The team erred on the side of incorporating all suggestions. However, we did not incorporate some changes that would make the content too specific to 1 country in the region rather than identifiable across several countries and did not incorporate changes that would be better served in future adaptations of the book (e.g., instructions for specific herding techniques).

#### Translation

Local country teams provided translations of the text, which was initially drafted in English. The design team then produced high-resolution PDFs of these translations for printing, digital distribution, or physical presentation.

#### Presentation and Distribution

*Living Safely With Bats* was made available to all project countries and partners, other interested users, and the general public. Some countries expanded beyond the original book design by reformatting the content for display on overhead projectors, easels, and posters, further demonstrating the book’s adaptability.

### Building a Visual Narrative

**FIGURE 3:**
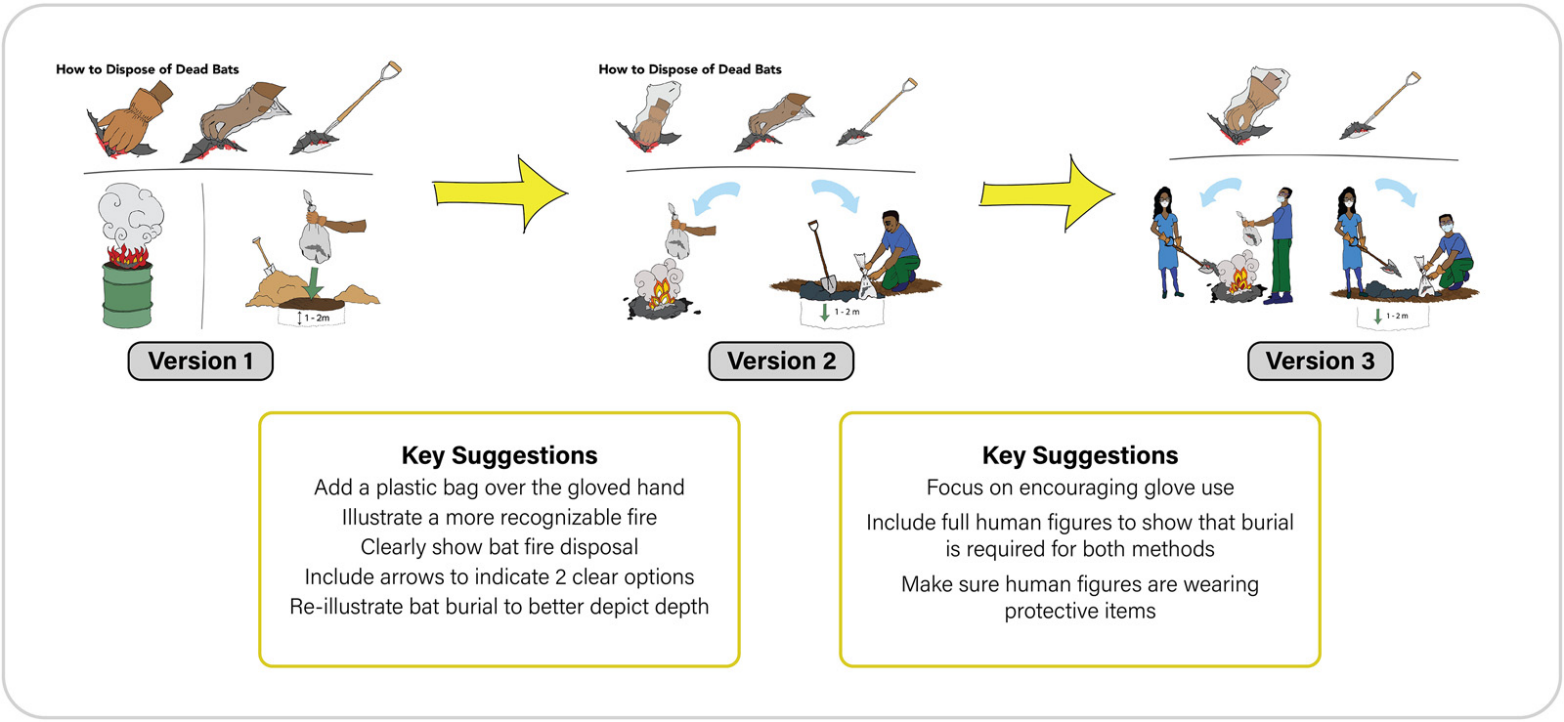
Incorporating Stakeholder Feedback Through an Iterative Content Development Process for Living Safely With Bats Book

*Living Safely With Bats* needed to be accessible to individuals with varying levels of literacy and knowledge regarding topics such as routes of disease transmission. As such, the artwork had a critical role to play in conveying these messages. The design team created a visual story from the talking points and created corresponding illustrations for each page using an iterative process in close consultation with the subject matter experts to ensure the accuracy and clarity of messages. Figure 3 shows 3 versions of the same page with suggested revisions from reviewers incorporated into each iteration.

Because the book needed to be accessible to individuals with varying levels of literacy and knowledge, the artwork had a critical role to play in conveying public health messages.

**FIGURE 4:**
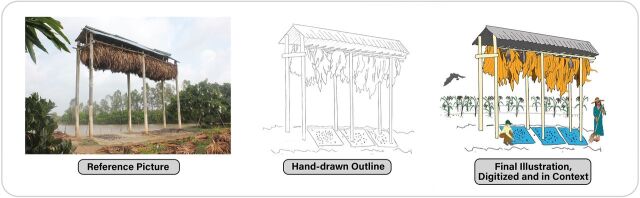
Example of Progression From Reference Photo of Guano Harvesting Structure in Vietnam^a^ (A) to Hand-Drawn Outline (B) to Final Illustration (C) to Create Images Used in Living Safely With Bats Book ^a^Photo credit: 2013 PREDICT Vietnam/Wildlife Conservation Society.

Technical experts provided the artists with reference images of building structures, natural features, local flora and fauna, and colors and designs of objects that would be immediately recognizable. Images were created first as unfilled line drawings that were sketched with pen and pencil on white paper. Illustrations were then scanned and uploaded for digital editing (Figure 4). All artwork was cleaned and converted into vectors using Adobe Photoshop and Illustrator and then laid out using InDesign (Adobe Inc.). Examples of locally inspired or informed illustrations include the following.

In Section 1, the talking points discuss the critical role bats play in eating insects that can damage crops. In an initial version of the corresponding illustration, a bat was shown in a 2-panel illustration catching a nonspecific gray-winged insect in its mouth over a field of generic stalks with colorful flowers. The adjacent panel showed an alternative scenario where the overabundance of these insects without bat control caused them to wilt. After feedback from country partners, the crops were redesigned to be corn, and the insects were changed to depict beetles and grasshoppers. In the version used for distribution in Asia, the corn was replaced with rice fields.

In Section 2, the water container and food tray cover were inspired by images of the versions of these receptacles used throughout West Africa.

The version of the book distributed to partners in Asia included durian flowers because bats pollinate this fruit, which is highly valued in the region.

The book aimed to avoid negative attributions or stereotypes of bats, particularly in light of communities’ proximity to bat populations, as well as researchers’ desire to retain a species conservation message. To achieve broad applicability, the book depicted bats in a manner that made them indistinguishable from particular species. The pages that addressed the dangers of bat contact avoided using exaggerated or cartoonish features such as fangs. Careful consideration was given in the text to ensure that bats were not characterized as frightening disease carriers, with attributions being made instead to infectious fluids of infected bats. These considerations aimed to balance the bat conservation message alongside harm reduction messaging and avoid fear-based rhetoric in conveying risk communication messages.

### Using a Moderated Storytelling Format

*Living Safely With Bats* is divided into the following 6 modules that address the topics identified as most relevant by local stakeholders and subject matter experts.

1. Bats Are an Essential Part of Our Ecosystem
2. Ways to Live Safely With Bats: The Basics
3. How to Dispose of Dead Bats
4. If Contact With Live Bats Is Unavoidable
5. Managing Bats in the Home
6. Managing Bats Around the Outside of Your Home

Trusted community leaders, such as village elders and community health agents, were identified to best fulfill the moderator role as they serve as powerful local actors in combating rumors and disinformation, which were recurring themes in our behavioral findings.^16^ Having local leaders as moderators also ensures that the knowledge shared in the book remains in the community even after the project’s close.

Trusted community leaders, such as village elders and community health agents, were identified to best fulfill the moderator role as they serve as powerful local actors in combating rumors and disinformation.

The book was designed to be printed in a landscape format with binding along the longer top edge, with talking points facing the moderators and a corresponding full page of text-free illustrations facing the audience. While the provided talking points for each module were written as a full script, the book is intended to drive robust discussion about local mitigation strategies that could be implemented to reduce the risk of zoonotic disease transmission.

The modular format allows moderators to adapt the content to different presentation contexts (e.g., short meetings or multiday workshops), risk compositions (e.g., hunting communities or communities with or without bat tourism), as well as tailor the discussions to local contexts and languages. Moderators could rearrange or omit sections depending on the context and constraints within which they were working.

### Adapting Content for Other Regions

Although *Living Safely With Bats* was initially designed for use in West Africa, the team further developed the content to be used with project partners throughout East, South, and Southeast Asia. In addition to using adapted artwork, the version used in Asia included 2 additional modules that were informed by consortium partners (particularly in Cambodia, Vietnam, Myanmar, and Malaysia) who shared insights regarding local practices around guano- and bat-related tourism. These 2 additional modules were: (Module 7) If Contact With Bat Feces Is Unavoidable (to address practices around bat guano collection) and (Module 8) Basic Ways to Protect Yourself in Caves and Other Bat Sites (to address bat ecotourism and religious tourism in bat cave sites).

## IMPLEMENTATION FINDINGS AND DISCUSSION

We review lessons learned from the process of developing *Living Safely With Bats*, including recommendations and potential impact in future use.

The first immediate application of *Living Safely With Bats* was conducted in advance of the public announcement of the discovery of BOMV in Sierra Leone. Starting in July 2018, the project teams worked closely with ministry officials and other public health stakeholders in Sierra Leone, and in Liberia and Guinea in August and September 2018, respectively, to provide scientific guidance on the discovery and to mobilize the book as a resource to combat misinformation. These included collaborations with Ministries of Health and Sanitation (Sierra Leone), Agriculture and Forestry (Sierra Leone), Higher Education and Scientific Research (Guinea), Health (Guinea), Environment (Guinea), Livestock (Guinea), Health (Liberia), and the National Public Health Institute of Liberia.

The PREDICT Liberia team rehearsed facilitating questions around the book’s content and the new virus findings so that they would be prepared to address any concerns or rumors sparked by the announcement. After this effort, the team traveled to several communities to present *Living Safely With Bats* and to encourage community conversation around practical risk mitigation strategies. In Sierra Leone, the presentation of *Living Safely With Bats* was rolled out to 27 of the 30 project communities.^15^

The book was also used as a part of community trainings in Sabah, Malaysia, and Peninsular Malaysia. More than 800 attendees participated in these events and over 100 books were distributed in 2 languages to community leaders and schools.

**FIGURE 5:**
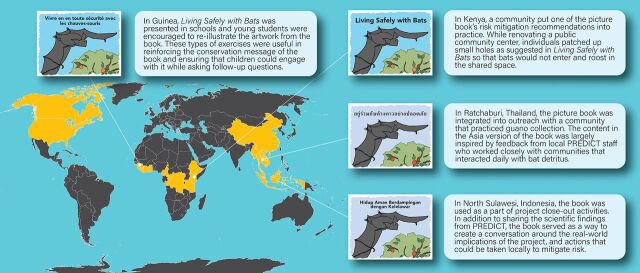
Map of Distribution of Living Safely With Bats in 20 Countries and How It Was Used in Guinea, Indonesia, Kenya, and Thailand

The book was translated into 12 languages and rolled out in more than 20 countries (https://p2.predict.global/living-safely-with-bats-book). Figure 5 shows how the book was used in Guinea, Indonesia, Kenya, and Thailand.

A formal program evaluation was not feasible due to the timing of the BOMV finding and the overall PREDICT project coming to an end. However, our process for developing a multidisciplinary, community-focused One Health tool may provide insights to others who need to develop similar resources within the context of an EID public health emergency, particularly regarding coronaviruses.

### Lessons Learned

#### Decide Early on How to Integrate Feedback

A recurring topic of conversation during the development of *Living Safely With Bats* was whether to take either a gold standard or harm reduction approach to mitigation suggestions.^17^^–^^19^ Because many of the places where the book would be shared were communities with limited access to running water and personal protective equipment, several experts suggested providing information regarding “safer” practices instead of dictating that communities abstain from certain behaviors, such as those associated with their livelihoods. Ultimately, the decision was made to err on the side of encouraging a gold standard approach, with an understanding that community discussions would naturally include perspectives on both ends of the spectrum. This highlights the crucial role of the moderator in the book’s implementation. As feedback conflicted at times depending on whether the reviewer followed a harm reduction or gold standard philosophy, future adapters can save time by deciding early on which ideology the book will adapt.

Moderators have a crucial role in implementing the book by deciding early to use a gold standard or harm reduction approach to adapting mitigation suggestions.

#### Anticipate Adaptations and Invest in a Robust Tracking Infrastructure

Since collaboration and resource sharing are core competencies of a One Health approach, the project expected that the book would be made available for use in new contexts. For example, Breakthrough ACTION used the foundation of *Living Safely With Bats* to make a separate One Health educational resource called *Living Safely With Animals*. However, it became difficult to track the usage of the book as the process for obtaining it changed. At first, users were asked to complete a simple online submission form before obtaining a downloadable copy of the original version of *Living Safely With Bats*. An initial count recorded more than 100 unique users having downloaded the West Africa English version of the book. But with the creation of multiple language versions, increase in demand, and fulfillment of ad hoc requests, the various versions of *Living Safely With Bats* were eventually hosted on multiple platforms and made available for download as an open-access resource. Anecdotally, we know that the books were shared widely through email among professional networks and listservs. Although this distribution is consistent with the open-sharing philosophy of the project, future practitioners may find value in early planning and budgeting for a method of hosting, distributing, and measuring the use of the book. A centralized record of the decisions made for local adaptations would also allow practitioners to build from each other’s work and benefit from previous lessons learned.

### Key Recommendations

#### Incorporate Multidisciplinary, Multilevel, and Multicountry Contributions and Collaboration at Every Step

First, we recommend building in the expectation of multisectoral contribution and communication from the onset and into every step of the project. The book’s inception, topical coverage, and cohesion were made possible because of multisectoral representation in every facet of the project. We often received feedback from groups of experts in animal health, human health, and conservation. This dynamic was replicated from feedback received at the executive leadership level and from local country research teams. As a feature of the umbrella project, this multisectoral involvement ensured that a One Health lens was applied throughout the development process, rather than only at the end.

We recommend building in the expectation of multisectoral contribution and communication from the onset and into every step of the project.

#### Train Moderators to Convey Key Messaging While Adapting the Content for the Context

Second, significant time should be spent training moderators in mastering the scripted talking points so that they can modify their communication style as required. While this approach builds in flexibility to address community-specific needs and supports the moderator’s ability to communicate effectively with the audience, it also presents a chance for deviations from the book’s messaging. As such, for future practitioners, careful attention should be paid during moderator training to review the science behind each topic so that there is an understanding of each takeaway message. Mock group presentations can help identify sections of the book where moderators may need additional support or guidance. This recommendation also relates to the need for the project to decide on a harm reduction versus gold standard approach. Sharing this decision with moderators during the training may help these moderators in planning strategies to share information that stays true to the key messaging and answers questions.

#### Plan for the Unexpected

Third, we recommend that similar One Health research programs build in funding support and staff time from the beginning in anticipation of educational activities, both expected and unexpected. This will, at the very least, create an avenue for sharing research results, and at the most, create a path to accommodate a prolonged evaluation schedule including tool development; piloting; and pre- and posttests measuring changes in knowledge, attitudes, and behaviors. Because *Living Safely With Bats* was created in reaction to a public health urgency, time was a limiting factor in implementing a formal evaluation. Against the backdrop of greater global awareness of disease outbreaks and sobering predictions for the public health challenges that may lie ahead, it is prudent for any research project in the areas of One Health and pandemic prevention to build in science communication as a part of their project deliverables.

#### Conduct Monitoring and Evaluation of a Program Implementing the Book

Lastly, an evaluation of a program using *Living Safely With Bats* would be a boon for the evidence base around One Health and pandemic prevention–related behavioral interventions. Emerging scholarship is building the case for a contextually embedded understanding of knowledge, attitudes, and behaviors regarding zoonotic diseases and for related health education programs informed by a One Health approach.^20^^,^^21^ Future work could include a formal evaluation of the use of the book to measure impact and to gather insights on how to improve it to successfully achieve behavior change.^22^ We propose process and outcome eval-uations of interventions designed around using this book. These evaluations would measure the extent to which the intervention operates as intended and its effectiveness in changing knowledge, attitudes, skills, behaviors, and health related to living safely alongside bats.

### Potential Impact

Prevention strategies such as educational tools and behavioral interventions are essential actions for pandemic mitigation. Stopping zoonotic disease spillover is the most cost-effective and timely strategy for the pressing challenges of pandemics like COVID-19, over other postemergence strategies such as vaccines and drug therapies.^23^ During the global roll-out of *Living Safely With Bats*, stakeholders involved in the dissemination of the materials expressed an interest in creating books to address behaviors concerning exposure to other taxa associated with zoonotic diseases—most notably rodents and nonhuman primates. Assessing the ability of the book *Living Safely With Bats* to change knowledge, perceptions, and behavior could provide One Health practitioners with a roadmap on how to engage local stakeholders and the science community in building a toolkit to better protect human, animal, and environmental health.

## CONCLUSION

*Living Safely With Bats* is an educational tool that was developed to mitigate infectious disease spillover from bats. The process of developing this book shed light on a multistakeholder and multisectoral model that One Health practitioners can use when creating resources for zoonotic disease transmission mitigation with communities living in close contact with wildlife. The COVID-19 pandemic illustrates the opportunity to more widely distribute and expand on EID risk mitigation tools, as well as to create additional resources to address topics such as contact along rural-to-urban commercial wildlife supply chains, the harvesting of wildlife, and urban wildlife interfaces.

## Notes

**Peer Reviewed**

**First published online:** December 5, 2022.
